# Tumor Biomechanics-Inspired Future Medicine

**DOI:** 10.3390/cancers16234107

**Published:** 2024-12-07

**Authors:** Yuqing Dong, Mengnan Lu, Yuting Yin, Cong Wang, Ningman Dai

**Affiliations:** 1 The Key Laboratory of Biomedical Information Engineering of Ministry of Education, School of Life Science and Technology, Xi’an Jiaotong University, Xi’an 710049, China; 2 Bioinspired Engineering and Biomechanics Center (BEBC), Xi’an Jiaotong University, Xi’an 710049, China

**Keywords:** tumor biomechanics, tumor mechanobiology, tumor mechanomedicine

## Abstract

Malignant tumors pose a significant global health challenge, severely threatening human health. Statistics from the World Health Organization indicate that, in 2022, there were nearly 20 million new cancer cases and 9.7 million cancer-related deaths. Therefore, it is urgently necessary to study the pathogenesis of cancer and explore effective diagnostic and treatment strategies. In recent years, research has highlighted the importance of mechanical cues in tumors, which have become a new hallmark of cancer and a key factor in regulating tumor behavior. This suggests that studying the mechanical properties of tumors may open potential new avenues for understanding the pathogenesis, diagnosis, and therapeutic intervention of cancer. This review summarizes the mechanical characteristics of tumors and the development of tumor diagnostics and treatments targeting specific mechanical factors. Finally, we propose new ideas and insights for the application of mechanomedicine in cancer diagnosis and treatment in the future.

## Simple Summary

Physical factors play an important role in the development of cancer. From the perspective of tumor biomechanics and tumor mechanobiology, mechanical interactions in tumor microenvironment at multiple scales are clearly presented. For clinical needs, the typical examples in tumor mechanodiagnosis and tumor mechanotherapy are exhibited. Faced by the challenges of equipment and in vivo experiments, we considered employing artificial intelligence to predict and study tumor behaviors, as discussed at the end. For wider audiences including tumor researchers and clinicians, in this review, we put forward the concept of tumor mechanomedicine to introduce and summarize the relevant progress in recent years.

## 1. Introduction

The latest statistics from the World Health Organization indicate that in 2022 [1], there were nearly 20 million new cancer cases and nearly 10 million cancer-related deaths worldwide. It is projected that by 2050, the number of new cancer cases per year will reach 35 million, which is a 77% increase compared to 2022. The high incidence and mortality rates of cancer, coupled with the diversity of cancer characteristics, once again underscore the necessity for a global upgrade in targeted cancer control measures. With the increasing depth of research in biology and mechanics and the rapid development of related technologies, biomechanics became an independent discipline, studying the mechanical issues of life processes [2]. An increasing number of mechanical models are being applied to explain biological phenomena [3,4]. With the advancement of biomechanical research, mechanobiology has emerged and gradually become a highly regarded interdisciplinary field within the realm of biomedical engineering. In recent years, with the advancement of research on tumor-related biomechanics and mechanobiology, it has become possible to analyze the pathogenesis of tumors using their mechanical properties and to develop new diagnostic and treatment strategies [5]. However, there is still a lack of in-depth understanding of the biomechanical characteristics and the potential value in therapy of the mechanical properties of tumors during the process of tumor occurrence and development.

**Figure 1:**
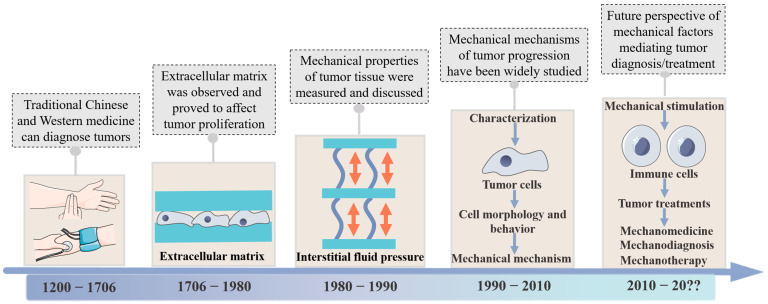
Illustration of the exploration of tumor biomechanics, mechanobiology, characteristics and mechanisms in different times. From this process, the typical diagnosis technique and research discovery are exhibited, including feeling the pulse for early tumor diagnosis, investigating the extracellular matrix and interstitial fluid pressure in tumors for biomechanics, revealing the mechanical mechanism in tumor progression by mechanobiology, and providing the tumor mechanomedicine strategy for future tumor treatments.

In this contribution, we firstly overview the development timeline of research on tumor mechanical factors (Figure 1). Then, relevant research works are summarized systematically to elucidate the mechanical properties of tumors and their impact on tumor behavior from the perspectives of tumor biomechanics and mechanobiology. The explorations of potential applications of mechanical properties in tumor diagnosis and treatment are presented next. Further, the challenges that lie ahead, in terms of tumor mechanical diagnostics and therapeutics are discussed. This review put forward the concept of “tumor mechanomedicine” which aims to provide a new research perspective and solution for the diagnosis and treatment of tumors.

## 2. Tumor Biomechanics and Mechanobiology

### 2.1. Tumor Biomechanics

Tumor biomechanics involves the comprehensive application of mechanical theoretical methods and experimental techniques to quantitatively study the mechanical characteristics of tumors at different scales (such as organ, tissue, cell, and molecular levels), and to reveal the mechanical mechanisms that affect the occurrence and development of tumors.

**Figure 2:**
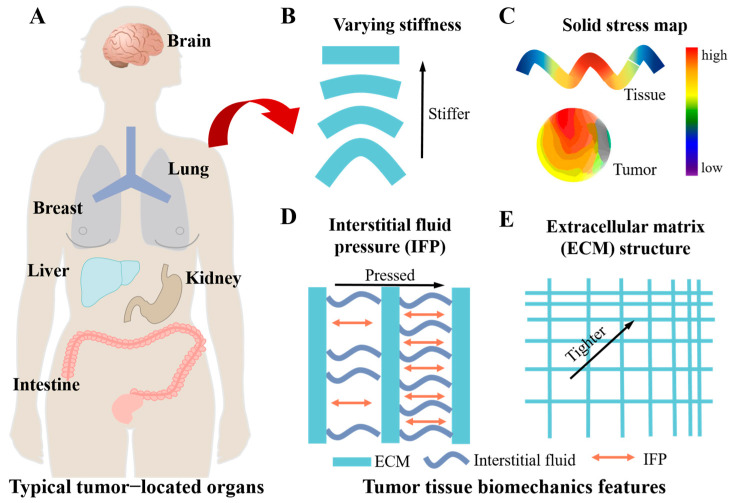
Characteristics of tumor biomechanics in organ and tissue scale. (**A**) A sketch of the human body with marked typical tumor-located organs including brain, lung, breast, liver, kidney and intestine. (**B**) Varying stiffness in tumor tissues. (**C**) Profile of solid stress in a tumor. (**D**) Increased interstitial fluid pressure in a tumor. (**E**) Different extracellular matrix structures in tumor tissues.

In organ and tissue scale, a tumor secretes cytokines and interacts with surrounding tissues during growth which can directly affect the structure of the organ, thereby altering the organ’s stiffness and viscoelasticity [6,7]. This indicates that the mechanical properties of tumors themselves are significantly different from normal tissues. Tumor location in these organs, such as brain, breast, liver, lung and intestine, always possesses typical tumor biomechanical characteristics including stiffness, solid stress, interstitial fluid pressure (IFP) and extracellular matrix (ECM) structure (Figure 2). For instance, the average stiffness of breast cancer and liver cancer is much higher than that of normal tissue [8,9]. The 2D map of solid stress in a brain tumor exhibits a radialized profile and local concentration [10]. Many solid tumors show an increased IFP, which forms a barrier in tumor treatment [11]. There are a number of factors that contribute to increased IFP in tumors, such as vessel abnormalities, fibrosis and contraction of the interstitial matrix. For example, the increased leakage of newly formed blood vessels within tumor tissue compresses the lymphatic vessels, which leads to the accumulation of interstitial fluid in the tumor, ultimately leading to an increase in IFP within the tumor tissue [12,13]. The growth and invasion of tumors disrupt the normal structure of ECM, and the local inflammatory microenvironment leads to excessive deposition and cross-linking of collagen, hyaluronic acid, and other ECM components synthesized by tumor cells and fibroblasts, causing the ECM structure of tumors to be tighter and remodeled [8]. As deposition and cross-linking of ECM increase, the local viscoelasticity of tumor tissue changes, and the density and stiffness of ECM significantly increases [6].

Apart from the tumors themselves inducing changes in mechanical characteristics through progression, non-cancerous cells, including fibroblasts, myofibroblasts and endothelial cells in tumor microenvironments, are also active in influencing the tumor tissue’s mechanical characteristics through cell–cell interactions. For example, cancer-associated fibroblasts (CAFs) are “educated” by cancer cells to secrete signaling molecules to stimulate the cells’ secreting ECM components and matrix-degrading enzymes and then remodel the ECM structure [14]. The CAFs’ behavior supports the malignant progression of tumors by regulating the mechanical characteristics of tumors. Matrix metalloproteinases (MMP), known as typical proteolytic enzymes, can degrade the components of ECM in tumor microenvironments, which leads to changes in ECM structure and stiffness [15]. Evidence has proven that MMP help profile and remodel the fiber networks in ECM to regulate the tumor invasion, neoangiogenesis, and metastasis behaviors. Especially in cancer progression, MMP is involved in the regulation and processing of adhesion and cytoskeletal proteins, growth factors, chemokines and cytokine [16]. Further, the structure changes of ECM by MMP can also be employed to benefit the ideal pharmacologic targets for cancer therapy.

**Figure 3:**
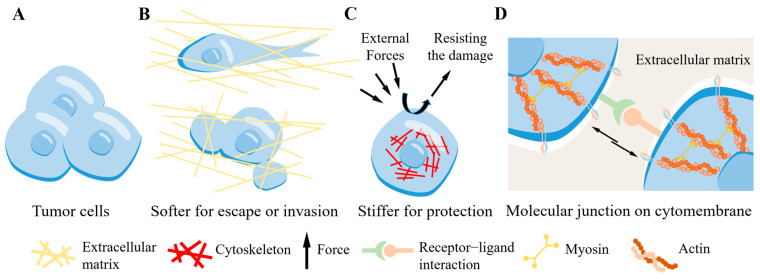
Characteristics of tumor biomechanics at the cell and molecular scales. (**A**) Normal tumor cells. (**B**) Soft tumor cell for escape/invasion. (**C**) Stiff tumor cell for self-protection. (**D**) Force junctions between cells by mechanically affected molecules and proteins.

At the cellular scale, tumor cells also possess unique mechanical characteristics (such as stiffness and viscoelasticity). As a tumor develops, the cellular-scale mechanical characteristics also continue to change [17]. A brief schematic diagram to introduce the physical properties at the cellular and molecular scales are shown in Figure 3. For example, compared with normal cells, tumor cells, such as breast cancer cells and melanoma cells, have lower stiffness. When tumor cells gradually invade, their stiffness and loss modulus will further decrease [18]. In contrast, circulating tumor cells (CTCs) can increase their stiffness by regulating the cytoskeleton to resist the damaging effects from fluid shear forces [19]. When migrating, durotaxis refers to the directed movement of melanoma cells and NIH3T3 cells along a gradient of stiffness [20,21]. These results suggest that tumor cells may alter their own stiffness as mechanotaxis to seek an environment which is similar to the proliferative environment they originally inhabited to enhance their proliferative capacity.

At the molecular scale, molecular clutches are molecular mechanical force transmission networks composed of integrins, talin, ECM, and the cytoskeleton. Clutches are used to transmit the interaction forces between tumor cells and ECM, and they mediate various biological behaviors such as tumor cell adhesion and migration [22]. Based on the characteristics of force transmission by molecular clutches, various mechanical theoretical models have been used to describe the force transmission process between tumor cells and ECM [23,24]. Cell–cell adhesion is mediated by cadherins, which attach to the actin cytoskeleton, thereby tightening intercellular connections [25]. Integrin-mediated focal adhesion complexes and cadherin-mediated adherens junction complexes can synergistically regulate cell functions under mechanical stimulation [26]. After sensing external mechanical signals, the mechanical signal transduction affects the adhesion, proliferation, invasion, and metastasis of tumor cells [27]. The physical connection between cytoskeleton and cell nucleus can also directly transmit mechanical signals, leading to changes in epigenetic and transcriptional information within the nucleus [28].

In summary, the mechanical characteristics of tumors differ significantly from those of normal tissues at various scales. These mechanical properties play a key role in the occurrence, progression, and drug resistance of tumors. Therefore, employing these differences in mechanical characteristics can provide new strategies for understanding tumor pathological features, early screening, diagnosis, and monitoring therapeutic efficacy.

### 2.2. Tumor Mechanobiology

**Figure 4:**
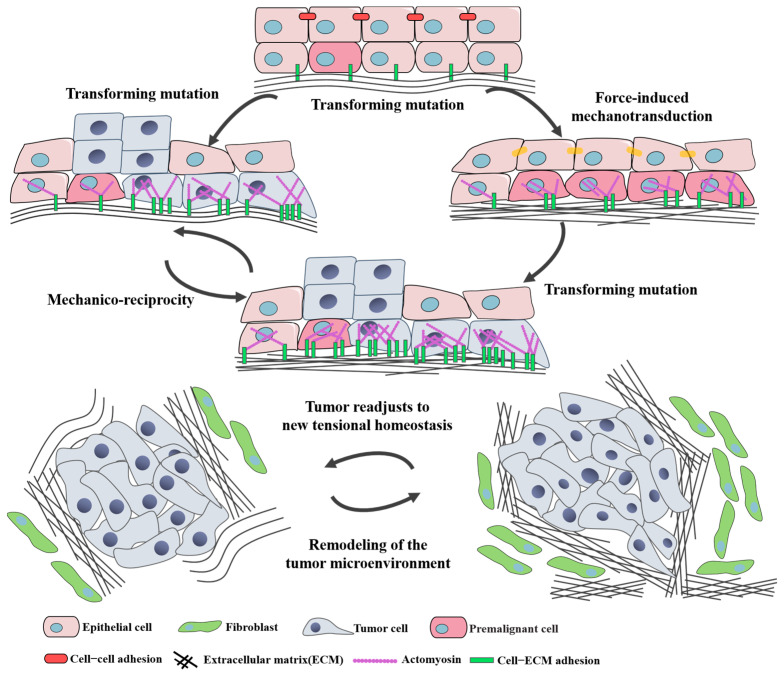
Illustration of tumor mechanobiology-based interactions between cells and ECM. The malignant transformation of epithelial cells occurs by the biomechanical property changes in the ECM, and then fibroblasts lead to the increased ECM stiffness and structural changes through the mechanotransduction which promotes the occurrence of malignant tumors. Further, through cell–cell/cell–ECM interactions, ECM is remodeling in the tumor microenvironment.

Tumor mechanobiology is targeting to explore the impact of mechanical microenvironment on the occurrence and development of tumors. Firstly, the initiation, distribution and transduction of mechanical force in cell–cell and cell–ECM interactions in the tumor microenvironment are presented in Figure 4. The following section describes the relationship and regulation manner between mechanical properties and tumor behaviors.

The biomechanical property changes in ECM pave the way for the malignant transformation of epithelial cells [29]. The increase in ECM stiffness and structural changes driven by fibroblasts are key factors in the occurrence of malignant tumors which are considered as biomechanical markers for the formation of tumors. The stiffness and solid stress of a tumor are regarded as mechanical factors that regulate tumor signaling pathways through mechanotransduction processes, thereby controlling tumor proliferation and invasion [30,31]. For example, increased ECM stiffness can promote tumor cell proliferation through the ERK signaling pathway [32], and it can also promote epithelial-mesenchymal transition (EMT) and enhance the invasive and migratory abilities of tumor cells through the mechanotransduction of integrins and downstream focal adhesion kinase (FAK) [33]. During the distant metastasis process, high fluid shear stress can cause cell cycle arrest and induce apoptosis in the majority of CTCs [34], while low fluid shear stress enhances the migration and invasion capabilities of CTCs [35]. Continuous tumor cell proliferation in a confined space certainly leads to prominent tissue pressure and stretch on cells, which can trigger the stretch-activated Piezo channel [36]. In Gudipaty’s work, they found stretching leads to cell division and crowding leads to extrusion, which can help determine how Piezo1 differentially interprets crowding versus stretching to become a tumor suppressor or not [37]. Additionally, the association between Piezo1 expression and the aggressive glioma phenotype is consistent with increasing tissue pressure, and Piezo1 upregulation and function amplify the level and speed of tissue stiffening to aggravate tumor progression [38]. However, the impact of differences in mechanical properties, such as stiffness and viscoelasticity, between different organs on the biological behavior of metastases has been less reported and remains to be further explored [39].

**Figure 5:**
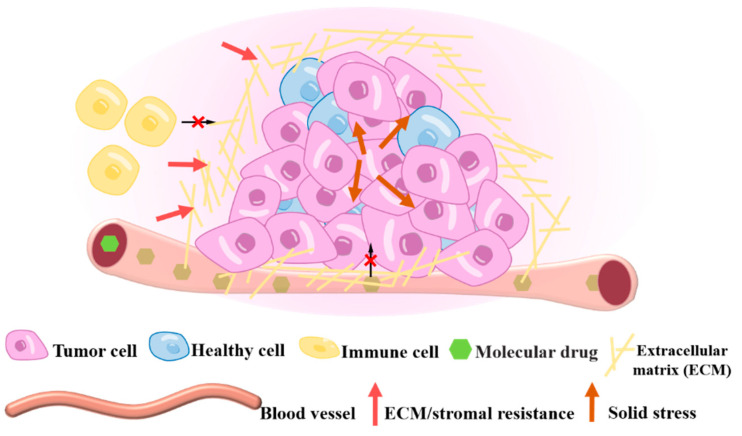
Illustration of tumor mechanobiology based on stimuli response to resist immune cell and drug therapy. The ECM becomes stiffer to resist immune cell invasion in the tumor tissue core; the ECM structure remodeling prevents the delivery of drugs in high-pressed blood vessels.

To avoid apoptosis or damage by external mechanical factors, the “smart tumor” has reacted a series of protection behaviors (Figure 5). For example, the mechanical characteristics of tumor tissue can hinder drug delivery, induce drug resistance to tumor cells, and restrain the infiltration and function of immune cells. Firstly, an abnormal ECM structure and increased IFP are considered the main factors that hinder drug delivery. A large amount of collagen deposition and cross-linking lead to an abnormally hard and dense matrix barrier, significantly limiting the ability of therapeutic drugs to reach the tumor [40]. Changes in ECM components, increased matrix stiffness, and integrin-mediated cell–cell adhesion can all lead to tumor cells escape from chemotherapy drugs [41]. Tumor cells are also stimulated to produce more immunosuppressive factors by activating signaling pathways such as YAP-TAZ with PDZ-binding motifs when experiencing the increase of ECM stiffness and solid stress [41,42]. In addition, the irregular cytoskeleton and soft cell membrane reduce the overall stiffness of tumor cells, weakening the killing function of T cells [43]. Moreover, the high IFP in the center of the tumor hinders the infiltration of T cells, leading to resistance to immunotherapy [44]. These mechanical characteristics, either alone or in combination, eventually ensure the tumor progression without immunization or drug therapy.

Cancer cells can show mechanical memory features in vitro [45,46], which is closely related to tumor metastasis [47]. Recently, Runx2-mediated mechanical memory has been found in bone metastasis of in vivo breast cancer [48]. The high stiffness of the primary tumor microenvironment (PTM) can promote activation of the ERK-Runx2 signaling axis, which continues to exist when they metastasize to the secondary tumor microenvironment (STM). The authors termed this process “soil instructs soil”. However, Runx2-mediated mechanical memory might be erased via proliferation (cell division). In addition, pancreatic cancer cells can be mechanically maintained to exert higher traction even when they are transferred to a softer matrix later [46]. For therapy, cancer cells respond to chemotherapy or targeted drugs differently, depending on the stiffness of their microenvironment [49]. In addition, for different breast cancer cell lines, the reduced therapeutic effect of drugs induced by increasing matrix stiffness is different. Since the stiffness of PTM and STM is distinct, such a difference could have important effects on therapeutic effects. If cancer cells have in vivo memory, mechanical memory behavior should also be considered in the treatment plan.

In summary, mechanical factors play a key role in the occurrence and development of tumors. A deep understanding of the impact of tumor mechanobiology on the diverse behaviors of tumor cells is conducive to elucidating the mechanisms of tumor pathogenesis and drug resistance, guiding the diagnosis and treatment of tumors.

## 3. Tumor Mechanodiagnosis

Compared to normal tissues, tumor tissues exhibit significant differences in mechanical properties such as stiffness, solid stress, IFP, and ECM structure. Therefore, measuring the mechanical properties of tumors and targeting mechanotransduction molecules is expected to become an effective means for early diagnosis of tumors, differentiation of benignancy and malignancy, and prediction of therapeutic efficacy. Tumor mechanodiagnosis builds upon existing methods such as imaging and pathology to further detect tumor mechanical characteristics or mechanotransduction molecules related to tumors, thereby achieving the goals of disease prediction, differential diagnosis, and prognostic stratification.

**Figure 6:**
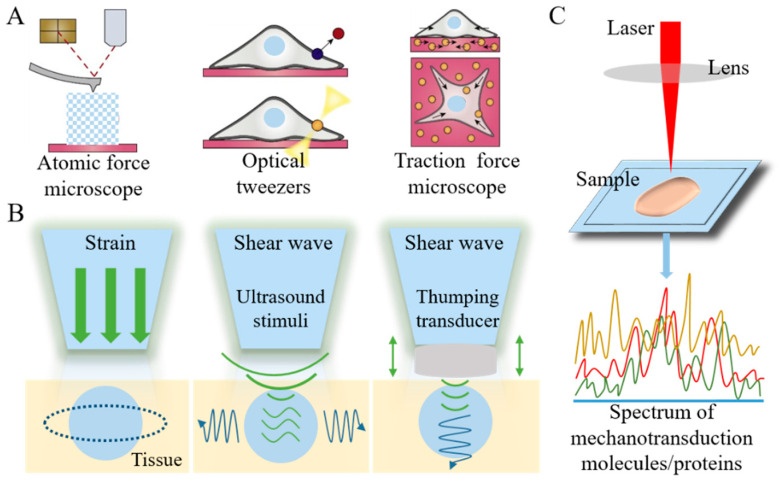
Illustration of tumor mechanodiagnosis techniques. (**A**) Force characterization methods including atomic force microscope, optical tweezers and traction force microscope. (**B**) Ultrasound elastography and magnetic resonance elastography by strain/stress imaging and wave imaging. (**C**) Biomedical spectroscopy to detect the spectrum of mechanotransduction molecules and proteins.

The materials and methods of typical tumor mechanodiagnosis-related technique or equipment need to be exhibited first (Figure 6). Due to intratumor heterogeneity, the differentiation of mechanical characteristics between tumor and normal tissues by in vivo cancer detection is difficult. Thus, in vitro mechanical characterization devices like AFM (atomic force microscope) is firstly required to measure and compare the modulus and viscoelasticity of tumor and normal tissues [50]. Optical tweezers and traction force microscope as force manipulation triggers can assist to characterize the force interactions in the tumor microenvironment at cell (cell traction force) and molecular (mechanotransduction protein) scales [51]. The outputs of these tumor mechanodiagnosis results are always basic physical parameters. For in vivo detection, tumor mechanodiagnosis by imaging, like the elastography technique, is now widely applied. Due to the differentiation of mechanical properties caused by varying structure, components and water contents, the in vivo diagnosis of tumors by elastography becomes visualized. In addition, pathological detection by biomedical spectroscopy techniques for tumor mechanodiagnosis is also important, which can observe the morphology and structure of tumor tissue as well as the secretion of mechanosensitive proteins. Detailed progress in these fields is presented below.

### 3.1. Tumor Imaging Mechanodiagnosis

The most important imaging method currently used to detect the mechanical properties of tumor tissues is elastography. It assesses tumor mechanical properties by applying stress to the tumor and analyzing the resulting tissue deformation, which mainly involves ultrasound elastography and magnetic resonance elastography. Ultrasound elastography was the earliest technology used to measure tissue stiffness to assist in the diagnosis of tumors. Magnetic resonance elastography generates tissue viscoelastic images by analyzing the shear wave information produced by an external vibration source [52]. Compared with ultrasound elastography, magnetic resonance elastography provides higher spatial resolution and soft tissue contrast, making it suitable for detecting the viscoelasticity of deep tumors such as those in the brain [53]. It should be pointed out that elastography can reflect the water content of tumor tissue to help reflect ECM status. For example, determining the differences in water content between pancreatic tumors and normal pancreatic tissue as well as other organs can accurately quantify metabolic differences when using the water signal for normalization [54]. The vibrational photoacoustic imaging technique was recently developed to image water content, which overcame a limitation in deep tissue imaging [55]. Differing from healthy tissue, the parameters of IFP, fluid velocity and flow in tumors are also significant for clinicians to diagnose [56]. Numerous clinical studies have confirmed the outstanding performance of elastography in the diagnosis and differential diagnosis of breast cancer, thyroid cancer, liver cancer, and endometrial cancer. For example, conventional ultrasound and ultrasound contrast combined with real-time elastography can improve the detection rate of thyroid papillary carcinoma [57]. In addition, the combination of elastography data and deep learning ultrasound radiomics data can better predict the extent of lymph node metastasis in early breast cancer [58].

Molecular imaging technology is currently an important imaging method for detecting the mechanical properties of tumors at the molecular scale. Integrins are key molecules in mechanotransduction and are widely involved in various biological behaviors of tumors, making them potential targets for tumor diagnosis and staging. A variety of integrin receptor imaging agents have entered the clinical stage, among which radionuclide imaging based on integrin αvβ3 and αvβ6 is widely used for tumor diagnosis and efficacy prediction [59]. For instance, a prospective study showed that an 18F-labeled tracer targeting αvβ3 integrin can be used to detect recurrence in patients with glioblastoma multiforme, demonstrating good safety and diagnostic efficacy [60]. In addition to integrins, collagen has also been proven to be helpful for tumor diagnosis. By comparing the differences in collagen fiber imaging of different thymic tumors, diseases such as thymoma and thymic squamous cell carcinoma can be effectively distinguished [61].

### 3.2. Tumor Pathological Mechanodiagnosis

In addition to imaging methods, pathological sections can also be used to observe changes in tumor tissue morphology, structure, and ECM components, thereby assisting in tumor diagnosis and prognosis assessment. For instance, the expression level of type I collagen is an important indicator for predicting lung cancer bone metastasis and is negatively correlated with the survival of lung cancer patients [62]. Hyaluronic acid scoring can serve as an indicator for assessing the prognosis of pancreatic ductal adenocarcinoma, and for those with liver metastasis, a higher hyaluronic acid score is associated with lower overall survival (OS) and progression-free survival [63]. Analyzing changes in tissue structure on pathological sections can further assist in tumor diagnosis and prognosis assessment. Based on the morphological differences at the interface between the tumor and surrounding liver tissue, liver metastatic carcinoma can be classified into different growth patterns. Samples from liver metastasis patients with longer OS exhibit a pattern in connective tissue growth [64].

The diagnosis of differences in the expression of mechanotransduction molecules in tumors and tumor microenvironments is also studied. For example, integrin α7 is associated with vascular invasion, later Barcelona staging, and shorter OS in patients with hepatocellular carcinoma, while positive expression of integrin α3 is an independent risk factor for poor prognosis in stage IV non-small cell lung cancer patients [59]. In addition, FAK [65], piezo-type mechanosensitive ion channel component 1 [66], and others are also related to the occurrence and prognosis of various tumors. Characterizing these mechanosensitive proteins is expected to provide strong evidence for the auxiliary diagnosis of tumors, risk prediction, treatment guidance, and improvement of prognosis [67].

## 4. Tumor Mechanotherapy

In recent years, tumor mechanotherapy targets the mechanical characteristics of tumors at different scales, intervenes in the biological behaviors of tumor cells and their microenvironment, and thereby achieves the goal of killing tumors or enhancing the responsiveness of tumors to existing treatment methods. This therapeutic strategy aims to directly apply mechanical stimulation to eliminate tumors or target key molecules involved in ECM formation and mechanotransduction coupling to inhibit tumor progression. In addition, tumor mechanotherapy based on mechanical characteristics can be combined with traditional anti-tumor treatment strategies to further improve efficacy.

### 4.1. Tumor Single-Modality Mechanotherapy

Ultrasound and magnetic fields are widely considered means of directly intervening in mechanical characteristics at the tissue scale [68]. High-frequency low-intensity pulsed ultrasound and shockwave therapy aim to exploit the differences in mechanical properties between tumor cells and normal cells to achieve specific killing. Compared with normal cells, tumor cell nuclei have different inherent vibration frequencies. By precisely tuning these frequencies, high-frequency pulsed ultrasound can selectively destroy tumor cells without damaging healthy cells. An in vivo experimental result showed that shockwaves selectively killed malignant tumor cells in the body and inhibited tumor growth, which may be related to the increase in tumor cell membrane permeability and changes in nuclear mechanical properties (such as Young’s modulus) caused by shockwaves [69,70]. Magnetic fields can directly apply mechanical stimulation (such as compression, stretching, and twisting) to tumor cells by exerting magnetic forces on magnetic materials. For example, sharp magnetic materials adsorbed on the cell membrane can exert pressure and tension on the local cell membrane under the action of a rotating magnetic field, reducing the integrity of the cell membrane and thereby inducing tumor cell necrosis [68,71].

Therapy at the molecular-scale mechanical characteristics of tumors mainly focuses on key molecules involved in ECM formation and mechanically related molecular pathways. It is worth noting that targeting these key molecules may affect multiple tumor-related mechanical factors [72]. In terms of reducing ECM generation, studies have developed nano-delivery systems targeting FAP to inhibit excessive ECM deposition, enhance T cell infiltration, and promote tumor regression [73]. In addition, some inhibitors or monoclonal antibodies that inhibit the secretion of components such as collagen in the ECM are also being evaluated for their therapeutic efficacy. For example, TGF-β monoclonal antibodies, transforming growth factor-β receptor 1 (TGF-βR1) antagonists, and galectin-3 inhibitors can achieve anti-tumor effects by reducing the activation of CAFs and ECM cross-linking [73]. Studies have shown that inhibiting the activity of lysyl oxidase (LOX) can improve the structure of ECM, significantly reduce the degree of ECM fibrosis, and has great potential for tumor therapy [74,75]. Vascular endothelial growth factor (VEGF) is an important factor that controls angiogenesis and lymphangiogenesis, and its overexpression leading to tumor vessel leakage is an important reason for the increase in tumor IFP. At present, bevacizumab targeting VEGF has been widely used in the treatment of various tumors such as metastatic colorectal cancer [76]. Targeting collagen and hyaluronic acid treatments can refer to targeting the ECM and targeting IFP. Losartan, an angiotensin receptor inhibitor, can reduce solid stress by relieving the compression of blood vessels by hyaluronic acid and collagen, enhancing the delivery of drugs and oxygen [77]. FAK inhibitors (defactinib) and ROCK inhibitors (fasudil) have also been proven to reduce solid stress and fibrosis in tumor tissue by reducing the activity of CAFs [78,79]. CAFs are the main source of cells that cause ECM fibrosis, and TGF-β plays a core role in this process. Pirfenidone is a clinically approved TGF-β inhibitor that can significantly reduce the stiffness of the ECM in pancreatic cancer mice, while increasing vascular perfusion in the tumor to improve drug delivery and delay tumor progression [80].

### 4.2. Tumor Multimodality Mechanotherapy

The abnormal mechanical characteristics of tumors are considered to be important reasons for chemotherapy resistance, hindering drug delivery, and forming immunosuppressive microenvironments. Therefore, targeted regulation of these mechanical characteristics may not only exert therapeutic effects independently but also combine with chemotherapy, radiotherapy, and immunotherapy, thereby significantly improving the overall efficacy of anti-tumor treatment.

At present, the combination of drugs targeting tumor mechanical characteristics with chemotherapy has become a research focus. For example, anti-angiogenic drugs targeting tumor vasculature and VEGF, such as bevacizumab, in combination with chemotherapy, are considered a preferred strategy for normalizing tumor vasculature and reversing tumor drug resistance. A variety of novel LOX2 inhibitors or LOX inhibitors are being evaluated in early clinical trials, and their future combination with chemotherapy is expected to become a new method for anti-tumor treatment [81]. Furthermore, a Phase II clinical trial of neoadjuvant FOLFIRINOX (fluorouracil, irinotecan, and oxaliplatin) combined with losartan for patients with locally advanced pancreatic ductal adenocarcinoma showed that patients who received the combination therapy had a significant reduction in tumor stage and an increased rate of complete tumor resection [82]. A Phase I clinical trial of FAK inhibitors combined with gemcitabine and pembrolizumab for the treatment of patients with advanced pancreatic ductal adenocarcinoma also showed good safety and preliminary efficacy [83]. We know that the combined use of a β1 integrin antagonist and cisplatin has a better effect in stiff ECM, while the combination of EGFR and cisplatin has a better effect in soft tissues [84]. Therefore, when breast cancer invades bone tissue, it may be necessary to use EGFR and cisplatin in the beginning, and then a β1 integrin antagonist and cisplatin should be used to have better therapeutic effects.

The mechanical microenvironment of tumors plays a core role in the formation of immunosuppressive microenvironments. Normalizing tumor vasculature and remodeling the ECM or targeting mechanotransduction processes may enhance the response of tumors to immune checkpoint inhibitors and CAR-T cell therapy. Anti-angiogenic therapy targeting VEGF combined with immunotherapy has shown clinical effects in promoting T cell infiltration and drug penetration. ECM remodeling is closely related to the formation of immunosuppressive microenvironments, and targeting the TGF-β/TGF-βR1 pathway combined with immunotherapy can reverse the immunosuppressive microenvironment while remodeling the matrix. For example, M7824 is a bifunctional antibody targeting both TGF-β and the programmed death receptor-1/programmed death ligand 1 immune checkpoint pathways, which can significantly promote the activation of CD^8+^ T cells and NK cells, reduce tumor burden, and improve the survival of mice bearing breast cancer and colon cancer [85].

## 5. Prospects for the Development of Tumor Mechanomedicine

Tumor mechanomedicine has brought a series of revolutionary cognition and methods to the medical community. Mechanical factors have gradually proven to be of great significance in the pathogenesis, diagnosis and treatment of tumors, but there are still many challenges that need to be solved in this field.

**Figure 7:**
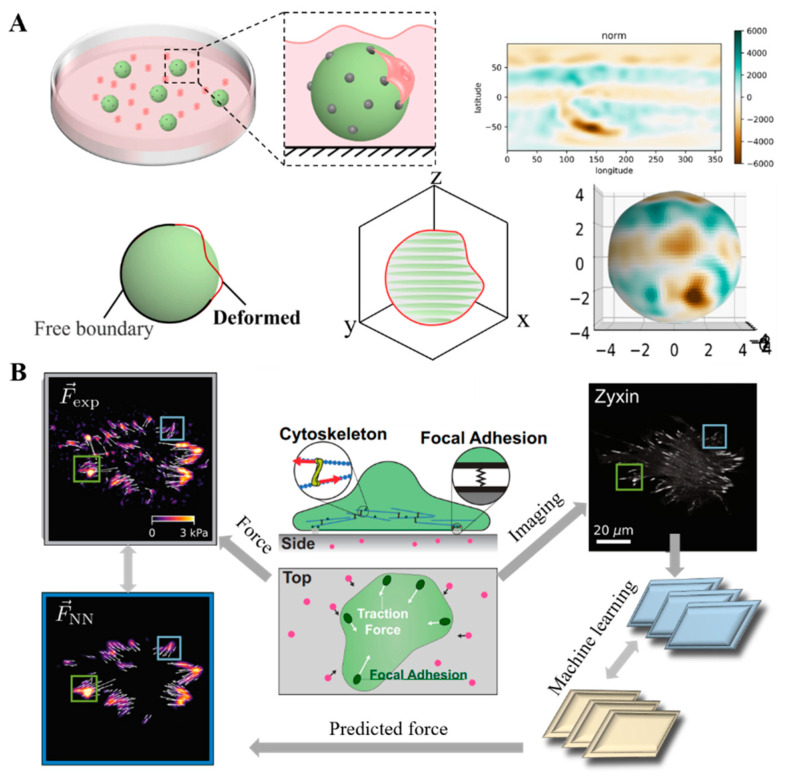
Prospects for the development techniques of tumor mechanomedicine. (**A**) Characterization of stress/force in the tumor microenvironment using an elastic microsphere sensor, the force-induced microsphere shape change can help calculate the stress fields. (**B**) Neural networks to predict cell traction forces by protein images using artificial intelligence; the focal adhesion and corresponding traction force in the sample are collected and tracked to further study and predict cell traction force.

Firstly, how to accurately measure the mechanical characteristics of tumors in vivo is an important problem that tumor biomechanics needs to overcome. Tools such as optical tweezers and AFM can obtain fine mechanical characteristics at the cellular and micro-tissue levels, but they are difficult to achieve in in vivo mechanical characterization of clinical patients. Using single-cell/molecular level mechanical probes [86] or wearable mechanical sensors [87] to construct an in vivo mechanical map is expected to provide effective mechanical information. The investigation of cell–cell interactions with fibroblast/CAFs or immune cells from a mechanical point of view is also promising. The basic contact and adhesion of CAFs or immune cells to tumors always exert force interaction which affects tumor growth, survival and malignant tendency [88]. On the contrary, cell force indexes also reveal CAFs or immune cell interaction and activity status when regulating the tumor microenvironment. Significantly, using elastic hydrogel microspheres as stress sensors can achieve the stress/force characterization for tumor biomechanics research at both the cell and tissue scales (Figure 7A) [89]. The mechanical characteristics of tumor tissue have undergone significant changes in the early stage of tumor development. The diagnosis and prognosis assessment of tumors through non-invasive means are also important in this field. Utilizing the distinction of the fluorescence lifetime (FLT) of tumor tissues by labeling with a biomarker (near-infrared dye) can achieve in vivo tumor imaging, which raises the efficiency of tumor diagnosis and resection [90]. This could inspire identifying the FLT of mechanosensitive proteins and help reflect the information of in vivo tumor tissue as one strategy for future tumor mechanomedicine.

Artificial intelligence can provide technical support for building multi-omics prediction models based on mechanical characteristics and is a key developmental direction for future tumor mechanodiagnosis (Figure 7B) [91]. Currently, targeting the ECM or intracellular mechanotransduction targets at the cellular and molecular scales is a hot direction for clinical translation. We need to develop more targets and perfect drug delivery carriers. In addition, the lack of technology that can directly intervene in the mechanical characteristics of tumors at the macroscopic level is another challenge faced by tumor mechanotherapy. Implanting hydrogels with specific mechanical properties to change the local mechanical microenvironment of tumors or directly applying forces to tumors through magnetic forces, ultrasound, mechanical devices, etc., is expected to further improve the efficacy of anti-tumor treatment.

## 6. Conclusions

Ultimately, the conception of “tumor mechanomedicine” and its related techniques are expected to be developed to finally be efficiently applied in clinical practice. Nowadays, liver stiffness is regarded as one index to distinguish malignant from benign tumors and serves as a cirrhosis prognostic marker. In bone and cardiovascular health, mechanobiological markers, including integrins and Piezo ion channels are proven to reveal disease states by responding to mechanical forces. For therapy, the checkpoint of mechanically induced immune cell activation and evasion as tumor mechanodiagnosis is also developing. In summary, the prosperity and development of tumor mechanomedicine not only depends on interdisciplinary cooperation among clinical oncology, biology, biomedical engineering, physics, and computer science but also requires continuous innovation and progress in technical methods. We sincerely hope that this study can reveal the key role of mechanics in the diagnosis and treatment of tumors to readers and further promote research and discussion in related fields.
